# Metabolic Syndrome and Selective Inflammatory Markers in Psoriatic Patients

**DOI:** 10.1155/2016/5380792

**Published:** 2016-12-21

**Authors:** Simona Vachatova, Ctirad Andrys, Jan Krejsek, Miloslav Salavec, Karel Ettler, Vit Rehacek, Eva Cermakova, Andrea Malkova, Zdenek Fiala, Lenka Borska

**Affiliations:** ^1^Department of Dermatology and Venereology, University Hospital Hradec Kralove, 50005 Hradec Kralove, Czech Republic; ^2^Institute of Clinical Immunology and Allergology, Faculty of Medicine in Hradec Kralove, Charles University in Prague, 50038 Hradec Kralove, Czech Republic; ^3^Department of Transfusion Medicine, University Hospital Hradec Kralove, 50005 Hradec Kralove, Czech Republic; ^4^Department of Medical Biophysics, Faculty of Medicine in Hradec Kralove, Charles University in Prague, 50038 Hradec Kralove, Czech Republic; ^5^Institute of Hygiene and Preventive Medicine, Faculty of Medicine in Hradec Kralove, Charles University in Prague, 50038 Hradec Kralove, Czech Republic; ^6^Institute of Pathological Physiology, Faculty of Medicine in Hradec Kralove, Charles University in Prague, 50038 Hradec Kralove, Czech Republic

## Abstract

The presented article studies the role of selected inflammatory and anti-inflammatory serum markers of psoriatic patients in the pathogenesis of metabolic syndrome (MS) and psoriasis. The study is based on the comparison between the group of psoriatic patients (74) and the control group (65). We found significantly higher BMI (*p* < 0.05) and diastolic blood pressure (*p* < 0.05) in the psoriatic patients. The values of waist circumference and BMI were significantly higher (*p* < 0.05) in the male patients compared to the men in the control group. The analysis revealed significantly higher CRP (*p* < 0.001), Lp-PLA2 (*p* < 0.001), leptin (*p* < 0.01), and resistin (*p* < 0.01) levels in the psoriatic patients. Significantly higher levels of CRP (*p* < 0.01), Lp-PLA2 (*p* < 0.001), leptin (*p* < 0.01), and resistin (*p* < 0.05) were found in the patients with MS compared to the controls with MS. The level of adiponectin was significantly lower (*p* < 0.01) in the patients with MS. Finally, we found significantly higher level of Lp-PLA2 (*p* < 0.001) in the group of patients without MS compared to the controls without MS. In conclusion, observed inflammatory and anti-inflammatory markers (CRP, adiponectin, leptin, resistin, and Lp-PLA2) are involved in both pathogenesis of MS and pathogenesis of psoriasis. The level of Lp-PLA2 indicates the presence of subclinical atherosclerosis (cardiovascular risk) in psoriatic patients.

## 1. Introduction

Psoriasis is a multifactorial chronic inflammatory disease with the prevalence of 2-3% in Europeans [1]. The pathogenesis of psoriasis is complex and the exact mechanism remains elusive. The disease is thought to result from a combination of genetic, epigenetic, and environmental influences. Psoriasis affects primarily the skin and/or joints and is characterized by keratinocyte hyperproliferation, angiogenesis, and immunopathological inflammation, which is clinically manifested under the picture of erythematous plaques with scaling on the skin [2, 3].

Recently, psoriasis has been recognized as a systemic disease associated with multiple comorbidities [4–6], including Crohn's disease, ulcerative colitis, diabetes, chronic neuropathy, depressions, lymphomas, multiple sclerosis, malignant processes, especially lymphoproliferation and nonmelanoma skin cancers, metabolic syndrome (MS) [7, 8], and cardiovascular diseases, such as hypertension, myocardial infarction, and stroke [9]. For comorbidity and mainly for the increased risk of cardiovascular disease, the risk of shortening the life of psoriatic patients is 5 years [10]. The systemic inflammatory state seems to be the common denominator for all these comorbidities [4].

The white adipose tissue is now recognized as the central player in the low-grade inflammatory state characterizing the metabolic processes such as obesity [11]. Adipose tissue and resident macrophages are the source of a number of secreted biologically active proteins; therefore, adipose tissue is recognized as an endocrine organ. These proteins are known as adipokines [12, 13]. Adipokines possess both effects, pro- and anti-inflammatory, and they act through autocrine, paracrine, and endocrine mechanisms. The unbalanced production of pro- and anti-inflammatory adipokines in obesity contributes to the development of a chronic low-grade inflammation state, which seems to favour worsening of psoriasis lesion [11]. Increased production of most adipokines in obese people has an impact on multiple functions such as appetite and energy balance, immunity, insulin sensitivity, angiogenesis, blood pressure, lipid metabolism, and haemostasis, all of which are linked to cardiovascular diseases.

Presented article studies the role of selected proinflammatory and anti-inflammatory serum markers in the pathogenesis of psoriasis and MS with special attention to the risk of atherosclerosis. From the group of adipokines, we chose adiponectin, leptin, and resistin. Adiponectin is highly expressed by adipocytes with potent anti-inflammatory properties. Low serum levels of adiponectin are associated with adverse metabolic states such as diabetes, metabolic syndrome, atherosclerotic cardiovascular disease, and psoriasis [14]. Leptin, a protein secreted by adipose tissue, plays important roles in metabolism and has proinflammatory effects. It activates monocytes and macrophages to produce proinflammatory IL-6 and TNF-*α*. Leptin also enhances the production of proinflammatory Th1 cytokines and suppresses the production of anti-inflammatory Th2 cytokines at the same time [15]. Resistin is another adipocyte-specific proinflammatory polypeptide synthesized mostly by macrophages and monocytes contained in fat tissue. It was named for its ability to induce insulin resistance [14].

Our group of adipokines was completed by determining of nonspecific inflammatory biomarker, C-reactive protein (CRP), which is one of the most important reactants of the acute phase. Metabolic inflammation increases the level of CRP which strongly correlates with the degree of obesity [16, 17]. For the atherosclerosis risk evaluation, we selected lipoprotein-associated phospholipase A2 (Lp-PLA2), which is also preferentially secreted by monocytes and macrophages [16].

## 2. Materials and Methods

### 2.1. Observed Groups

In total, 74 patients with psoriasis and 65 healthy blood donors (control group) were enrolled in this case-control study. Patients with psoriasis (PP), suffering from active plaque psoriasis not treated by systemic drugs, were hospitalized at the Department of Dermatology and Venereology, University Hospital Hradec Kralove (Czech Republic) in the years 2012–2014. The group consisted of 33 women and 41 men (average age 50.6 years, age range 18–80 years, 30 smokers and 44 nonsmokers). The subjects with insulin resistance, diabetes, and cardiovascular diseases (under medication) were included into the study; however, the subjects with infections or other inflammatory diseases were excluded. Our patients were not treated by any drugs influencing inflammatory reaction. Patients suffering from psoriatic arthritis were excluded from the study.

The control group (CG) consisted of 32 women and 33 men (average age 51,6 years, age range 20–65 years, 20 smokers and 45 nonsmokers). The CG consists of healthy blood donors (obtained from the Department of Transfusion Medicine, University Hospital Hradec Kralove). The subjects with insulin resistance, diabetes, and cardiovascular diseases (under medication) were included into the study; however, the subjects with infections or other inflammatory diseases were excluded. Also those who suffer from arthritis were excluded. The persons (CG) were not treated by any drugs influencing inflammatory reaction.

The study was approved by the Ethics Committee of the University Hospital in Hradec Kralove, Czech Republic. Informed written consent was obtained from each person in the PP and the CG.

### 2.2. Metabolic Syndrome, Body Mass Index, and PASI Score

Samples of peripheral blood were collected from the cubital vein from the patients with psoriasis and from the control group (BD Vacutainer sampling tubes). Blood serum was isolated by centrifugation and stored under −70°C until analysis. Repeated thawing and freezing were avoided. The blood samples were examined for the following: fasting glucose, high-density lipoprotein, and triglyceride (analyzed by standard method).

Metabolic syndrome (MS) was determined in the PP and in the CG by accepting the criteria for diagnosis of the MS, developed by the National Cholesterol Education Program Adult Treatment Panel (NCE/ATPIII) [18]. When a subject has three of the five listed criteria, a diagnosis of the metabolic syndrome can be made. The criteria listed include (1) glucose intolerance presenting higher fasting glucose ≥5.6 mmol/l or known treatment for diabetes; (2) increased waist circumference (WC) or abdominal obesity (≥102 cm for men and ≥88 cm for women); (3) raised triglyceride (TAG) levels ≥ 1.7 mmol/l; (4) reduced high-density lipoprotein (HDL) < 1.03 mmol/l for men and <1.30 mmo/l for women; and (5) elevated blood pressure (systolic blood pressure, SBP ≥ 130 mmHg, and/or diastolic blood pressure, DBP ≥ 85 mmHg).

Body mass index (BMI) was calculated as the ratio of weight and height squared (kg/m^2^). The state of disease was calculated from basic characteristics of actual disease status (erythema, desquamation, and skin infiltration) and expressed as the PASI score (Psoriasis Area and Severity Index) [19].

### 2.3. Selected Inflammatory Markers

The blood samples were examined for the following: C-reactive protein (CRP), adiponectin, leptin, resistin, and lipoprotein-associated phospholipase A2 (Lp-PLA2).

The level of CRP was assessed by immunonephelometry on IMMAGE 800 (Beckman, USA) and results were expressed in milligrams (mg) per liter of serum with detection limit of 1.0 mg per liter.

The level of adiponectin was detected by sandwich ELISA method using commercial kit Quantikine Human Total Adiponectin/Acrp30. The concentration of adiponectin was expressed in milligrams (mg) per liter of serum with detection limit of 0.0246 mg/l. Leptin was assessed by ELISA technique using kit Quantikine Human Leptin with detection limit 0.78 micrograms (*μ*g) per liter. Serum levels of resistin were determined using ELISA kit Quantikine Human Resistin with detection limit 0.026 nanograms (ng) per milliliter of serum. All kits were manufactured by R&D Systems (USA) and used according to the manufacturer's instructions.

The serum lipoprotein-associated phospholipase A2 (LpPLA2) concentrations were determined by a sandwich enzyme-linked immunosorbent assay (ELISA) technique using kit for Human LpPLA2 (Cloud-Clone Corporation, USA) according to the manufacturer's instructions. The limit of detection of the LpPLA2 was 0.263 nanograms (ng) per milliliter. Absorbance values were read at 450 nm using a Multiskan RC ELISA reader (Thermo Fisher Scientific, USA).

### 2.4. Statistical Analysis

The differences between the PP and CG were compared by two-sided *t*-test (for normally distributed data). For non-normally distributed data, we used nonparametric Mann–Whitney* U* or Kolmogorov-Smirnov test. Height, weight, and waist circumference were evaluated separately in men and women. The Kruskal-Wallis nonparametric analysis of variance and post hoc Dunn's multiple comparison test with Bonferroni modification were used to compare inflammatory levels in the patients and the controls with/without MS.

## 3. Results

In the group of 74 PP, 12 patients (16.2%) had mild psoriasis (PASI < 10), 44 patients (59.5%) had moderate (PASI 10–20) psoriasis, and 18 patients (24.3%) were diagnosed with the severe form of psoriasis (PASI > 20). Median (upper and lower quartile) of PASI score was 15.3 (19.9–12.0).

We found MS in 61% (*n* = 45) of PP: 25 men (56%) and 20 women (44%). In the CG, we found MS in 48% (*n* = 31): 17 men (55%) and 14 women (45%). The differences in the number of men and women between the groups were insignificant.

Table 1 summarizes demographic and laboratory findings in the patients and the controls. We found significantly higher BMI (*p* < 0.05) and DBP (*p* < 0.05) values in the PP compared to the CG. The values of WC (*p* < 0.05) and BMI (*p* < 0.05) in males with psoriasis (PP) were significantly higher than in males without psoriasis (CG).

Table 2 shows parameters of inflammation in the whole groups (PP and CG). We found significantly higher CRP (*p* < 0.001), Lp-PLA2 (*p* < 0.001), leptin (*p* < 0.01), and resistin (*p* < 0.01) values in the PP compared to the CG. The difference between adiponectin levels in the PP and the CG was insignificant.

Table 3 describes parameters of inflammation in PP and in CG with MS. We found significantly higher CRP (*p* < 0.01), Lp-PLA2 (*p* < 0.001), leptin (*p* < 0.01), and resistin (*p* < 0.05) in the PP with MS compared to the CG with MS. The level of adiponectin was significantly lower (*p* < 0.01) in the PP with MS.

Table 4 displays parameters of inflammation in the PP and in the CG without MS. We found significantly higher Lp-PLA2 (*p* < 0.001) in the PP without MS compared to the CG without MS. CRP, adiponectin, leptin, and resistin were insignificantly higher in the PP without MS.

Table 5 describes parameters of inflammation in PP with and without MS. We found insignificantly higher CRP, resistin, and Lp-PLA2 in the PP with MS compared to the PP without MS. The level of adiponectin was significantly lower (*p* < 0.001) and the level of leptin (*p* < 0.05) was significantly higher in the PP with MS.

## 4. Discussion

As it was mentioned before, the psoriasis is associated with several comorbidities. It is believed that 73% of psoriatic patients have at least one comorbidity [4].

Metabolic syndrome is a combination of factors of cardiovascular risks, including central obesity, increased blood pressure, glucose intolerance, and dyslipidemia [19, 20]. In the presented study, we found significantly higher diastolic blood pressure in PP (DBP, *p* < 0.05). Our findings were consistent with the results of other authors [19]. In a group of psoriatic patients with comparable average age (53.7 years), they found higher level of blood pressure.

The prevalence of the MS is increased in psoriatic patients and occurs in 40% or even 65% of them [20]. In our experimental groups, we found very high occurrence of MS (61% in the PP and 48% in the CG). This high level of occurrence may be partially attributed to the higher age of the respondents (average age of 51.6 years) and also to the fact that almost 85% of the patients were diagnosed with moderate and severe form of psoriasis.

Balci et al. 2010 demonstrated that visceral fat area (VFA) is increased in psoriatic patients and is associated with the presence of psoriasis [21]. They also demonstrated that VFA is associated with the presence of metabolic syndrome in patients with psoriasis. Therefore, the increased accumulation of visceral adipose tissue, which releases proinflammatory cytokines, is a potential mechanism linking psoriasis to its metabolic comorbidities and may be a major contributor to the unfavourable cardiovascular risk in psoriasis. It was also substantiated that overweight is an independent risk factor for developing psoriasis and that obesity may increase the risk more than twice [20]. In our study, we found significantly higher BMI levels (*p* < 0.05) in the PP when compared to the CG. Naito and Imafuku suggest that men are more likely to acquire psoriasis if they have mild obesity in middle or older age [22]. In our study, the average values of waist circumference and BMI of the male psoriatic patients were significantly higher than in the controls (*p* < 0.05). However, significant differences were not found in females.

It seems that the processes of initiation and development of psoriasis and overweight/obesity are associated with various forms of chronic inflammation. Moreover, Ryan and Kirby summarized that suppression of systemic inflammation in psoriasis could also reduce metabolic inflammation [23]. The presented study was focused on inflammatory and anti-inflammatory mediators accompanying the psoriasis which leads to metabolic dysfunction, obesity, and atherosclerosis.

Metabolic inflammation and excessive adipose mass (obesity) increase the level of nonspecific inflammatory biomarkers, such as CRP [24, 25]. The level of CRP can also serve as a marker of psoriasis severity. Patients with moderate and heavy psoriasis have significantly higher levels of CRP than the healthy controls [26–28]. In our study, we found significant elevation of serum CRP levels in the PP when compared to the CG (*p* < 0.001; Table 2). Vadakayil et al. 2015 described elevated levels of CRP in psoriatic patients with the metabolic syndrome in comparison with the patients without metabolic syndrome [29]. We also found higher yet insignificant level of CRP in the PP with MS compared to the PP without MS (Table 5). Our psoriatic patients with MS have an average level of CRP significantly higher than the controls with MS (*p* < 0.01; Table 3), while the difference in the mean levels of CRP between the patients without MS and the controls without MS was not significant (Table 4).

It is now well established that adipose tissue is not only involved in energy storage but also serves as an endocrine organ that secretes various bioactive compounds called adipokines [30, 31]. Adiponectin represents one of the typical representatives of adipokines. The adiponectin increases insulin sensitivity and displays antiatherogenic and anti-inflammatory effects [32]. Additionally, it has been proposed that adiponectin may play a protective role against the development of hypertension [33, 34]. Low levels of adiponectin are associated with adverse metabolic states such as diabetes, metabolic syndrome, atherosclerosis cardiovascular disease, and psoriasis [35]. Unfortunately, previously published data on adiponectin levels in patients with psoriasis are still inconsistent. We can see that most of the studies show significantly decreased serum adiponectin levels in these patients compared to the controls [25, 26, 35, 36]. However, some studies have reported no significant difference of adiponectin levels in the psoriatic patients compared to the healthy controls [37, 38] and some recently published works found even increased levels of adiponectin in the patients compared to the controls [20, 39–41].

When we compared our experimental groups (PP and CG) without distinguishing them as the persons with and without MS, the level of adiponectin in the PP was insignificantly decreased (Table 2). After the division into the group of patients with MS and the group of controls with MS, we found significantly lower level of adiponectin (*p* < 0.01) in the group of patients with MS (Table 3). When comparing the group of patients without MS and the group of controls without MS, we found in the group of patients without MS even higher (nevertheless insignificant) level of adiponectin (Table 4).

Another representative of the group of adipokines is leptin. The level of this protein is increased in obesity in proportion to the fat mass. It is well known that this hormone regulates the central nervous system to reduce food intake by regulating neuropeptides in the hypothalamus [42]. Regulatory function of leptin is lost in long-term obese individuals, in whom leptin resistance is detected [43]. Several studies found significantly higher leptin level in psoriatic patients in comparison with controls [25, 26, 44]. Similar results are shown in the presented study. We found significantly increased level of leptin in the psoriatic patients compared to the controls (*p* < 0.01; Table 2) and in the psoriatic patients with MS compared to the controls with MS (*p* < 0.01; Table 3). Although the patients without MS had also higher leptin level than the controls, the difference between the values was insignificant (Table 4).

High serum leptin levels may play a relevant role in obesity-associated cardiovascular diseases including atherosclerosis. Elevated serum concentration of leptin has been found in patients with cardiovascular risk factors and obesity status [15]. It has been suggested that leptin may be a marker of severity of psoriasis [20]. It is assumed that adiponectin and leptin may be the links between psoriasis and their comorbidities. They are associated with obesity and metabolic syndrome, which in turn may contribute to increased risk of psoriasis and its exacerbations [20]. In the presented work, the level of leptin was significantly higher in the PP with MS compared to the PP without MS (*p* < 0.05; Table 5). Likewise, we found the level of adiponectin was significantly lower in the PP with MS compared to the PP without MS (*p* < 0.001; Table 5).

Adipokine resistin is an important factor linking obesity with diabetes [45]. Besides its contribution to the insulin resistance, it has been shown that resistin can trigger a proinflammatory state by regulating various biological processes, thus contributing to inflammatory diseases [46, 47]. Macrophages are the main source of resistin and obesity causes a significant macrophage infiltration of visceral white adipose tissue [30, 42]. Resistin has also been reported to be expressed in chronic disease states, such as rheumatoid arthritis, atherosclerosis, obesity, diabetes, and inflammatory bowel disease [40, 48]. Several studies reported higher levels of serum resistin in psoriatic patients compared to controls [26, 36, 49]. Our results are in accordance with the aforementioned literature data. We found significantly increased level of resistin in the PP compared to the CG (*p* < 0.01; Table 2) and in the PP with MS compared to the controls with MS (*p* < 0.05, Table 3). The patients without MS had also higher resistin level than the controls; however, the difference was insignificant (Table 4). Scientific literature demonstrated that increased resistin levels in patients with psoriasis were independent of their obesity [14]. Comparable levels (nonsignificant difference) of resistin in our patients with MS and without MS (Table 5) support these conclusions.

Lipoprotein (a) is composed of a low density lipoprotein- (LDL-) like particle to which apolipoprotein is linked by a disulfide bond [50]. Lipoprotein (a) is considered as the risk factor for ischemic cardiovascular disease serving there as a potent DAMP (damage-associated molecular pattern) signal inducing the inflammatory response [16]. It is evidenced that the transition from the safe pattern to DAMP is caused by the content of oxidized phospholipids in the lipoprotein (a). Lipoprotein-associated phospholipase A2 (Lp-PLA2) enzyme is bound to several plasma lipoproteins including LDL and HDL [50]. Lp-PLA2 is responsible for the biodegradation of phospholipids in plasma lipoproteins. Previously thought Lp-PLA2 was producing abnormal phospholipids with atherogenic and proinflammatory potential. Indeed, Lp-PLA2 is elevated in patients with hypercholesterolemia [16, 50]. However, this enzyme is also able to catalyze the further degradation of already oxidized risky phospholipids [50]. To summarize, Lp-PLA2 is displaying Janus face. When bound to LDL lipoproteins, proatherogenic, proinflammatory, and anti-inflammatory activities are seen. When bound to HDL lipoproteins, Lp-PLA2 confers clear antiatherogenic protection with pronounced anti-inflammatory and antioxidative activity [50].

Lp-PLA2 is expressed in macrophages of atherosclerotic plaques and can serve as a biomarker of cardiovascular risk (CVD) [51–53]. Increased Lp-PLA2 activity is associated with MS and incidence of fatal and nonfatal CVD [54].

Unfortunately, there is still not enough information available regarding psoriasis and corresponding level of Lp-PLA2. We found two scientific articles describing increased level of Lp-PLA2 activity in patients with psoriasis [55, 56]. In accordance with these studies, we observed significantly increased level of Lp-PLA2 in the psoriatic patients compared to the controls (*p* < 0.001; Table 2), in the patients with MS compared to the controls with the MS (*p* < 0.001; Table 3), and in the patients without MS compared to the controls without MS (*p* < 0.001; Table 4). It should be noted that we found insignificantly higher level of Lp-PLA2 in the patients with MS compared to the patients without MS (Table 5). Holzer et al. observed that the activity of Lp-PLA2 was significantly increased in the psoriasis group [55, 56]. Interestingly, antipsoriatic (anti-inflammatory) therapy tended to recover Lp-PLA2 activities, suggesting the other antiatherogenic potentials [56]. All these facts support assumption about the presence of subclinical atherosclerosis (cardiovascular risk) in the patients with psoriasis, regardless of the presence of MS.

We would like to notice that the evaluation of the differences of inflammatory markers in the entire groups (PP CG, Table 2) was carried out with the knowledge that the numbers of people with MS are different in these groups. This follows from the literary findings, which indicate that the incidence of MS in PP is higher than that among the general population from which the CG was selected [20, 57].

Finally, we would like to do a short recapitulation. Rajappa et al. observed higher levels of proinflammatory adipokines (leptin and resistin) and lower levels of anti-inflammatory adipokines (including adiponectin) in patients with psoriasis [58]. Zhu et al. found significantly higher leptin level in the patients with psoriasis compared to the controls [59]. Li et al. reported significantly lower level of adiponectin and insignificantly higher level of leptin in psoriatic patients [60]. Coimbra et al. described significantly higher levels of leptin and CRP and significantly lower level of adiponectin in psoriatic patients [61]. We can say that the outputs of our study are consistent with the results of all aforementioned authors. From the results in Tables 3 and 4, it is apparent that the combination of MS and psoriasis significantly increases the expression of inflammatory and anti-inflammatory cytokines and increases the risk of CVD. The presented data support general opinion that cytokines produced by adipocytes (adiponectin, leptin, and resistin) are involved in the pathogenesis of MS as well as in the pathogenesis of psoriasis.

## 5. Conclusion

We have demonstrated that selected inflammatory and anti-inflammatory markers (CRP, adiponectin, leptin, resistin, and Lp-PLA2) are involved in both pathogenesis of metabolic syndrome and pathogenesis of psoriasis. In addition, the level of Lp-PLA2 indicates the presence of subclinical atherosclerosis (cardiovascular risk) in psoriatic patients.

## Figures and Tables

**Table 1:** Demographics and laboratory findings in the patients (PP) and the controls (CG).

Variable	PP (*n* = 74)	CG (*n* = 65)	*p* value
Age	50.4 (18–80)	51.6 (20–65)	NS
Gender (male : female)	41 : 33	33 : 32	NS
BMI (kg/m^2^)	28.6 (32.9–24.0)	26.8 (29.8–24.5)	<0.05
BMI (kg/m^2^) male	30.7 (37.7–24.8)	26.8 (30.0–24.5)	<0.05
BMI (kg/m^2^) female	27.5 (30.4–23.7)	26.8 (29.5–24.3)	NS
Waist circumference male (cm)	110 (128.5–93)	99 (107–93)	<0.05
Waist circumference female (cm)	94 (103.5–80.5)	85.5 (101.2–80.2)	NS
DBP (mmHg)	90 (91–80)	84 (92–77)	<0.05
SBP (mmHg)	140 (150–129)	140 (148–127)	NS
HDL male (mmol/l)	1.1 (1.40–0.9)	1.0 (1.3–0.9)	NS
HDL female (mmol/l)	1.2 (1.4–1.0)	1.1 (1.2–0.9)	NS
Triglyceride (mmol/l)	1.5 (2.2–1.0)	1.2 (1.7–0.9)	NS
Glucose (mmol/l)	5.8 (7.2–5.1)	5.6 (6.6–4.9)	NS

Notes: all data (except age) are presented as medians and upper and lower quartiles in brackets. The age is presented as an average and range in brackets; BMI: body mass index, DBP: diastolic blood pressure, SBP: systolic blood pressure, and HDL: high density lipoprotein.

**Table 2:** Inflammatory markers in the patients (PP) and the controls (CG).

Variable	PP (*n* = 74)	CG (*n* = 65)	*p* value
CRP (mg/l)	6.1 (9.2–2.6)	2.2 (3.8–1.4)	<0.001
Adiponectin (mg/l)	9.3 (16.1–4.8)	10.8 (19.1–6.9)	NS
Leptin (ug/l)	20.7 (34.7–8.3)	10.4 (18.1–4.9)	<0.01
Resistin (ng/ml)	11.7 (16.8–8.4)	9.6 (11.8–7.9)	<0.01
Lp-PLA2 (ng/ml)	999.7 (1244.5–732.5)	631.0 (790.5–424.0)	<0.001

Notes: all data are presented as medians and upper and lower quartiles in brackets; CRP: C-reactive protein, Lp-PLA2: lipoprotein-associated phospholipase A2.

**Table 3:** Inflammatory markers in the patients (PP) and the controls (CG) with metabolic syndrome.

Variable	PP with MS (*n* = 45)	CG with MS (*n* = 31)	*p* value
CRP (mg/l)	6.6 (11.0–2.9)	2.4 (5.4–1.5)	<0.01
Adiponectin (mg/l)	6.0 (11.7–4.1)	12.9 (19.5–7.7)	<0.01
Leptin (ug/l)	26.0 (38.1–12.4)	10.5 (20.6–5.7)	<0.01
Resistin (ng/ml)	11.8 (15.6–8.4)	9.5 (10.8–8.1)	<0.05
Lp-PLA2 (ng/ml)	1007 (1245–761)	636 (887–486)	<0.001

Notes: all data are presented as medians and upper and lower quartiles in brackets; CRP: C-reactive protein, Lp-PLA2: lipoprotein-associated phospholipase A2.

**Table 4:** Inflammatory markers in the patients (PP) and the controls (CG) without metabolic syndrome.

Variable	PP without MS (*n* = 29)	CG without MS (*n* = 34)	*p* value
CRP (mg/l)	3.9 (7.4–1.8)	2.2 (3.6–1.4)	NS
Adiponectin (mg/l)	13.4 (23.8–7.5)	9.7 (18.0–4.7)	NS
Leptin (ug/l)	12.1 (26.5–5.8)	9.2 (15.0–3.9)	NS
Resistin (ng/ml)	11.4 (20.3–9.3)	9.8 (12.7–7.9)	NS
Lp- PLA2 (ng/ml)	942 (1242–660)	579 (719–408)	<0.001

Notes: all data are presented as medians and upper and lower quartiles in brackets; CRP: C-reactive protein, Lp-PLA2: lipoprotein-associated phospholipase A2.

**Table 5:** Inflammatory markers in the patients (PP) with and without metabolic syndrome (MS).

Variable	PP with MS (*n* = 45)	PP without MS (*n* = 29)	*p* value
CRP (mg/l)	6.6 (11.0–2.9)	3.9 (7.4–1.8)	NS
Adiponectin (mg/l)	6.0 (11.7–4.1)	13.4 (23.8–7.5)	<0.001
Leptin (ug/l)	26.0 (38.1–12.4)	12.1 (26.5–5.8)	<0.05
Resistin (ng/ml)	11.8 (15.6–8.4)	11.4 (20.3–9.3)	NS
Lp-PLA2 (ng/ml)	1007 (1245–761)	942 (1242–660)	NS

Notes: all data are presented as medians and upper and lower quartiles in brackets; CRP: C-reactive protein, Lp-PLA2: lipoprotein-associated phospholipase A2.
